# Analysis of Ti- and Pb-based particles in the aqueous environment of Melbourne (Australia) via single particle ICP-MS

**DOI:** 10.1007/s00216-022-04052-0

**Published:** 2022-04-28

**Authors:** Raquel Gonzalez de Vega, Thomas E. Lockwood, Xiaoxue Xu, Claudia Gonzalez de Vega, Johannes Scholz, Maximilian Horstmann, Philip A. Doble, David Clases

**Affiliations:** 1 Institute of Chemistry, University of Graz, 8010 Graz, Austria; 2 The Atomic Medicine Initiative, University of Technology Sydney, 15 Broadway, Ultimo, NSW 2007 Australia; 3 School of Biomedical Engineering, University of Technology Sydney, 15 Broadway, Ultimo, NSW 2007 Australia; 4 Institute of Inorganic and Analytical Chemistry, University of Münster, Corrensstr. 48, 48149 Münster, Germany

**Keywords:** Lead, Nanoparticles, SP ICP-MS, Single particle analysis, Environmental interaction

## Abstract

The analysis of natural and anthropogenic nanomaterials (NMs) in the environment is challenging and requires methods capable to identify and characterise structures on the nanoscale regarding particle number concentrations (PNCs), elemental composition, size, and mass distributions. In this study, we employed single particle inductively coupled plasma-mass spectrometry (SP ICP-MS) to investigate the occurrence of NMs in the Melbourne area (Australia) across 63 locations. Poisson statistics were used to discriminate between signals from nanoparticulate matter and ionic background. TiO_2_-based NMs were frequently detected and corresponding NM signals were calibated with an automated data processing platform. Additionally, a method utilising a larger mass bandpass was developed to screen for particulate high-mass elements. This procedure identified Pb-based NMs in various samples. The effects of different environmental matrices consisting of fresh, brackish, or seawater were mitigated with an aerosol dilution method reducing the introduction of salt into the plasma and avoiding signal drift. Signals from TiO_2_- and Pb-based NMs were counted, integrated, and subsequently calibrated to determine PNCs as well as mass and size distributions. PNCs, mean sizes, particulate masses, and ionic background levels were compared across different locations and environments.

**Graphical abstract:**

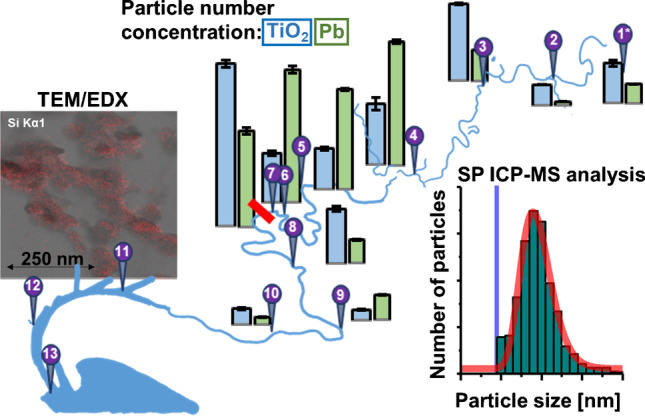

**Supplementary Information:**

The online version contains supplementary material available at 10.1007/s00216-022-04052-0.

## Introduction

Nanomaterials (NMs) have various properties that are dependent on their size, shape, and composition[1, 2]. They are ubiquitous in the environment, but their effects and functions in natural processes have often been overlooked or ignored due to a lack of analytical tools [2, 3]. NMs are produced on a large scale via natural pathways but also from incidental and intentional anthropogenic processes, and may be grouped into three categories describing their origin: natural, incidental, and engineered [3, 4]. Natural and anthropogenic global fluxes of NMs have been estimated to be hundreds of Tg (10^12^ g) per year [3]. The formation of natural NMs is a complex process that depends on numerous physical and chemical parameters that vary across different environments and times. Weathering, the abundance of precursors, dissolution, redox reactions, precipitation, and aggregation are some of these parameters and may affect the formation and stability of NMs [3]. Incidental NMs have been unintentionally produced since the Industrial Revolution by anthropogenic activities and their abundance may even exceed the levels of naturally occurring NMs in certain areas with high anthropogenic pressure. As new scientific and industrial applications for NMs have emerged in the last century, the production of engineered NMs has increased. Research applications include dedicated drug delivery systems [5], diagnostic imaging [6, 7] and therapeutic agents [5]. Industrial applications range from electronics, alternative energy sources [8], agriculture [9], coatings and paints [10].

Dedicated procedures are required to identify and further investigate the abundances, sizes, shapes, composition, and reactivities of NMs [2, 3] to understand their environmental behaviour and fate. While some NMs may have been vital in the geological history of the earth and even for the origin of life [3, 11–13], others may have an adverse impact on ecology and health [14]. Specifically, the abundance and effects of suspended NMs in surface waters and the oceans are highly relevant, but remain mostly unexplored. Furthermore, environmental systems are complex and the exact behaviours and reactions of NMs within environmental matrices are difficult to predict obscuring knowledge of types, rates, and extent of transformations as well as associated risks [15].

The analysis of suspended NMs in surface and oceanic waters requires analytical methods that are capable of analysing large sample sets and volumes in complex matrices, while providing efficient NM counting and models of elemental compositions, sizes and/or masses. Techniques like scanning (SEM) or transmission electron microscopy (TEM) with energy-dispersive x-ray analysis (EDX) are suitable to investigate the morphology and elemental composition of individual NMs but are limited for the determination of number concentrations and the construction of larger representative models of size distributions, and matrix- or particle-particle interactions. Surface processes and the charge of particles can be described using a zeta potentiometer, and the hydrodynamic diameter by dynamic light scattering and nanoparticle tracking. Further methods that are relevant for the characterisation of NMs include size exclusion chromatography and flow-field flow fractionation which were used to perform size separations. A summary and comparison of techniques for the characterisation of NMs can be found in a review by Mourdikoudis et al. [2].

Mass spectrometry has emerged as a promising alternative for the characterisation of NMs and, specifically, inductively coupled plasma-mass spectrometry (ICP-MS) enables advanced NM characterisation. ICP-MS was developed approximately 40 years ago and is a platform technology for the sensitive analysis of most elements of the periodic table. In ICP-MS, a hot Ar plasma is used as atomisation and ionisation source to produce elemental ions which are subsequently extracted, mass-filtered, and detected. The hyphenation of ICP-MS with secondary instrumentation such as chromatography and laser ablation (LA) has expanded the capabilities of ICP-MS further by providing speciation data [16] and spatial distributions of elements [17]. Application of rapid mass analysers enabled the detection of discrete particles when NMs are introduced into the plasma individually [18]. In this so-called single particle (SP) ICP-MS, each NM produces a spatially secluded ion cloud, which is extracted, analysed, and detected individually as a single pulse. The underlying methodology was introduced and gradually advanced over the last two decades [19–21], and was further applied to the analysis of micrometre-scaled particles like microplastics [22, 23]. Recent application to unicellular organisms has led to the development and refinement of methods collectively known as single cell ICP-MS [22, 24, 25]. The frequency of detected pulses is proportional to the number concentration of particles, and the pulse intensity is proportional to the targeted isotope mass. The introduction of rapid mass analysers enabled the reduction of dwell times from milliseconds to microseconds to detect extracted ion clouds from individual particles with several data points, thereby improving accuracy and signal to noise ratios [26–28]. The quadrupole has emerged as a frequently applied mass analyser, which however limits the number of investigated *m/z* to 1 per NM. An elegant and more recent technology to acquire multiple elements in a single NM is SP ICP-ToF (time-of-flight)-MS, which also offers rapid signal acquisition and improved mass resolution [29]. This is useful for screening NMs and in-depth characterisations [30, 31]. Praetorius et al. [32] and Loosli et al. [33] demonstrated that SP ICP-ToF-MS improved the characterisation of suspended NMs and were able to distinguish between natural and engineered NMs by comparing elemental compositions of particles. Similarly, Azimzada et al. [34] described the global abundance and composition of Ti-, Ce-, and Ag-based NMs. The performance of ICP-MS can also be improved via sector-field ICP-MS and ICP-MS/MS. While the former may increase mass resolution and as such improve the selectivity and sensitivity especially for interfered isotopes in SP analysis [35], it is often employed for its higher ion transmission rather than increased mass resolution [36, 37]. ICP-MS/MS may be used to exploit chemical affinities and reactions mitigating spectral interferences and improving figures of merit for interfered isotopes [38].

Altogether, SP ICP-MS offers unique strategies for the analysis and counting of individual NMs while providing high sample throughput. Dedicated models may also be constructed to describe number and size distributions, particle-particle interactions, and to differentiate between ionic and particulate analytes [39]. SP ICP-MS has previously been applied to investigate the levels and sizes of NMs in the environment. For example, Loosli et al. [33] analysed TiO_2_ NMs following sewage spills; Gondikas et al. [40] detected and quantified particles in surface waters; and Sanchis et al. [41] investigated the occurrence of CeO_2_, Ag, and TiO_2_ NMs in river water.

This study employed SP ICP-MS to investigate NMs in environmental waters surrounding Melbourne, a major metropolis in Australia. Samples were sourced from different locations in and around the city including rivers, ports, and lakes within proximity to major facilities such as airports, industrial grounds, and wastewater treatment plants. Aside from Ti as previously reported NMs, a screening method for SP ICP-MS employing a quadrupole mass analyser with a large mass bandpass was developed to pinpoint particulate elements in the high mass range. This procedure identified particulate Pb in various samples, and particle number concentrations (PNCs), size and elemental mass distributions, figures of merit, and the ionic background across the courses of major rivers were determined.

## Materials and methods

### Chemicals and consumables

Ultra-pure water was obtained from an Arium Pro system (Sartorius Lab Instruments GmbH & Co., KG, Germany). Elemental standards for ICP-MS were purchased at 10 μg mL^−1^ from Choice Analytical (Thornleigh, NSW, Australia). The detector deadtime was determined and compensated following the analysis of a diluted Er standard. The daily performance of the ICP-MS instrumentation was monitored and optimised by analysing a tuning solution containing 1 ng mL^−1^ Li, Y, Tl, Ce, and Ba. A 15 ± 1.3 nm Au NP dispersion (NanoXact Nanopheres—Bare, Citrate, 99.99 % purity) was obtained from nanoComposix (CA, USA) in 2 mM sodium citrate solution, stored at 4 °C and sonicated before each use. The characterisation of the Au NPs was undertaken by the manufacturer using ICP-MS and TEM. 0.45-μm PTFE syringe filters were obtained from Tisch Scientific (North Bend, OH, USA).

### Instrumentation

An 8900 series ICP-MS system (Agilent Technologies, Santa Clara, CA, USA) was equipped with platinum cones and s-lenses and operated with MassHunter software (Agilent Technologies). A Scott-type double-pass spray chamber (Glass Expansion, West Melbourne, Victoria, Australia) was cooled to 2 °C, and a MicroMist™ concentric nebuliser (Elemental Scientific, Omaha, NE, USA) was used for sample nebulisation. This study investigated samples obtained from river, surface, brackish, and coastal seawater in the catchment of Melbourne (Victoria, Australia). These complex environmental matrices were not immediately compatible with ICP-MS due to the high salt content which causes the deposition of matter on the interface and ion lenses. A high matrix introduction (HMI) accessory was previously found adequate to bypass these issues by performing on-line aerosol dilution between the spray chamber and the torch for robust analysis [22, 42] and was employed to bypass drift while analysing samples with high salt content. A method with an increased mass bandpass further referred to as bandpass mode was used to screen for particulate species in all samples. Further information on the development and application of the bandpass mode can be found elsewhere [39, 43–45]. TiO_2_, as frequently reported nanoparticulate entity, and particulate Pb, identified via the bandpass mode, were investigated using a quadrupole dwell time of 0.1 ms (Table S1).

A JEOL2200FS TEM equipped with a QUANTAX EDS was used to characterise the morphology and the elemental composition of NMs in selected samples. The accelerating voltage was 200 kV.

### Campaign and sample preparation

**Fig. 1:**
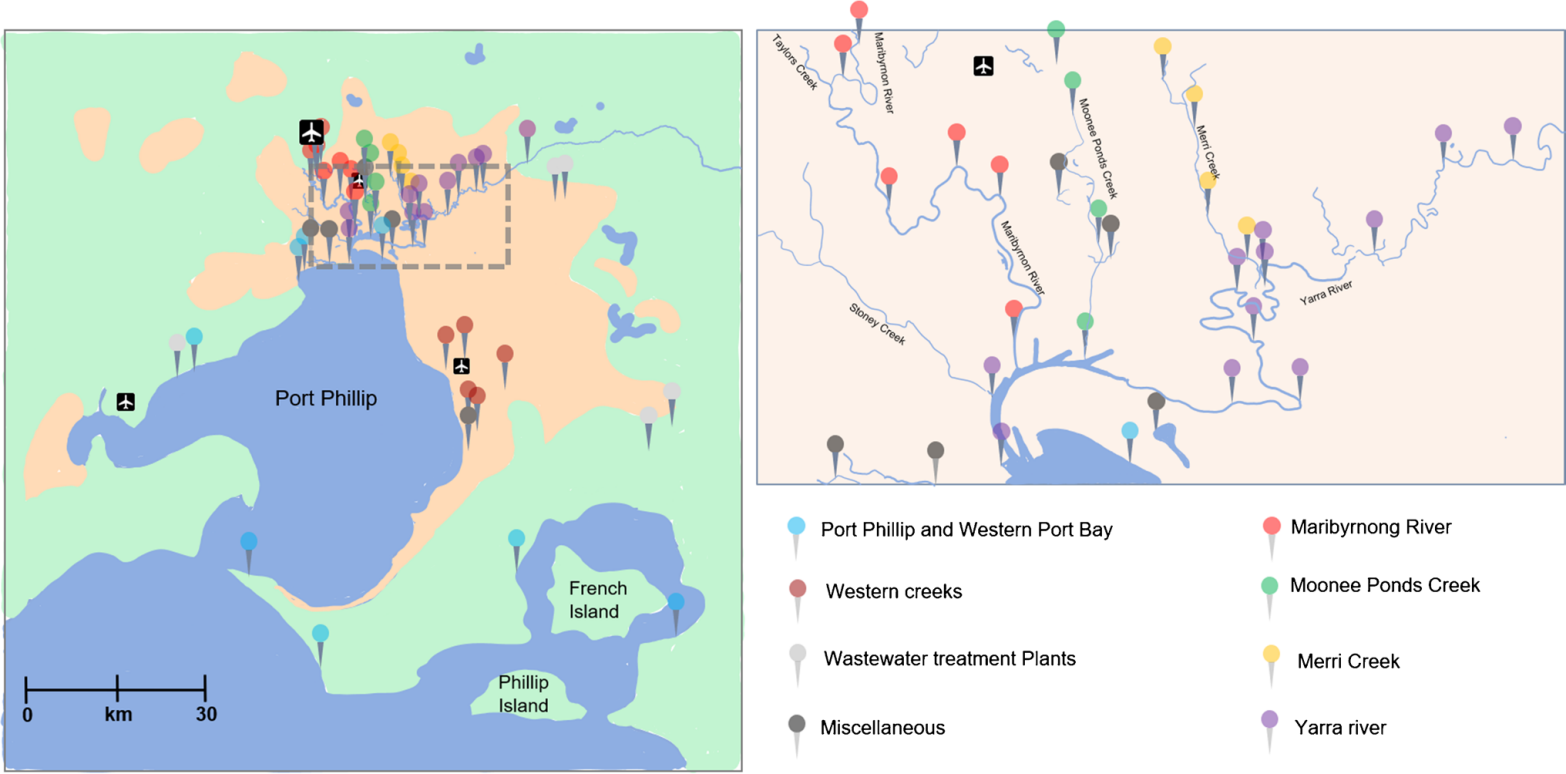
Sampling locations across the larger Melbourne area. Left: overview of all sample locations within the Melbourne area. Areas indicated in orange correspond to a high population/industrial density. The metropolitan area of Melbourne (dashed square) is magnified on the right and shows several rivers and the Port Philip estuary

The sampling campaign was undertaken from the 9th to the 11th of December 2019 within the catchment of greater Melbourne accommodating more than 5 million inhabitants (Figure 1). The sampling campaign time corresponded to Australian summer and temperatures deviated between 12 and 38 °C. There was no precipitation during the sampling period and no rain was recorded for more than 1 week prior. More information on the weather and tidal season is available in the supporting information (Figure S1). Samples were sourced at 63 different locations in polypropylene containers, frozen, and shipped within 24 h. The impact of freezing was evaluated by comparing the occurrence and mass of particles in representative samples before and after freezing and no significant differences were detected. Before sampling, containers where washed and passivated with the sampling matrix. Upon analysis, samples were thawed, sonicated in a water bath for 10 min at room temperature, and filtered (40 mL total volume) using syringe filters (0.45 μm pore size, PTFE) to remove larger particulate matter. The filtered water was immediately analysed via SP ICP-MS.

### SP ICP-MS data acquisition and processing

For the bandpass mode, the quadrupole was operated with a decreased mass resolution which enabled the analysis of several isotopes simultaneously. The bandwidth was tuned to collect any isotopes within mass windows of 8 amu using an acquisition time of 20 s. Six mass windows enabled single particle acquisition of a total of 25 elements with isotopes within the mass range of 139–204 amu in 2 min per sample. If signal spikes corresponding to individual NMs were detected in any mass window, nominal mass resolution was re-established in a second run for identification and further analysis.

Signal thresholding/recognition, accumulation, and calibration were performed with an in-house developed open-source Python-based data processing platform using the libraries PySide2, NumPy, and Bottleneck. Detailed information on thresholding algorithms and calibration pathways are available elsewhere [46]. Briefly, to distinguish signals of NMs from ionic background and noise, a dynamic Poisson filter was employed. This filter determined the mean signal over 1500 neighbouring data points and determined a threshold over which a signal was considered a NM. Limits of criticality and detection were calculated based on Poisson statistics for paired observations [26, 46, 47]. The transport efficiency (*η*) was estimated by analysing 15 nm Au NP dispersion and 1 ng/g Au ICP-MS standard diluted in ultra-pure and seawater using an uptake rate of 0.57 mL min^−1^. The transport efficiency was investigated in pure, sea, and river waters and no significant differences were recorded. Masses, sizes, PNCs, and figures of merit were calculated as reported previously [46].

## Results and discussion

### Method development and data analysis

Besides targeting Ti-based particles via SP ICP-MS, a bandpass mode was developed to screen for particulate elements in the high mass range. NMs containing elements within the high mass range have previously been described in the environment and may arise from a geogenic origin or anthropogenic discharges [41]. The screening for NMs in environmental matrices with a single collector instrument requires relatively large sample volumes and is time-consuming. To detect NMs at low PNCs, enough time must be spent on each *m/z* to collect sufficient particles containing the targeted isotope. To accelerate the screening process, the quadrupole mass bandwidth was tuned to be 8 amu and used to identify SP signal signatures from elements with isotopes within defined mass windows between 139 and 204 amu. Six mass windows (139–146 amu, 153–160 amu, 165–172 amu, 175–182 amu, 187–194 amu, and 197–204 amu) allowed screening for 25 elements in each sample. A total acquisition time of 20 s per mass window was used to detect signal spikes in samples. Samples with spikes were subsequently reanalysed with nominal mass resolution to identify the particulate element and to perform PNC, size, and mass calibrations. This approach enabled screening for high-mass elements in 63 samples within approx. 140 min. In addition to the acquisition of several *m/z* simultaneously, the bandpass mode also exhibited a higher ion transmission which improved the detection of small/light NMs as described elsewhere [39].

Water samples were collected along the course of major rivers in the Melbourne area. These rivers had estuaries in the Port Phillip Bay and transitioned from freshwater to brackish water, which complicated the analysis by SP ICP-MS. Complex matrices such as brackish water may be problematic due to the high abundance of matrix ions causing signal drift via space charge effects and salt deposition on the vacuum interface and ion lenses. The robust analysis of all samples with a common method required dilution to limit the salt burden in the plasma. One common strategy to mitigate matrix effects is the off- or on-line dilution with pure water. However, this may affect the stability of NMs, specifically the particle-particle and matrix-particle interactions. For example, changing the matrix composition may alter the adsorption of ionic analytes on the surface of NMs. Therefore, an on-line aerosol dilution system (HMI) was used to dilute already nebulised samples between spray chamber and plasma with an additional Ar gas flow. The robustness of this method was evaluated by repeatedly analysing and calibrating the size of an Au NP standard diluted in filtered coastal seawater over 90 min (compare Figure S2). The (size) drift rate was estimated to be 0.0354 % min^−1^ (compare Figure S2) using a linear fit and demonstrated the ability to perform robust analysis and calibration. However, the on-line aerosol dilution reduced the transport efficiency to between 0.14 and 0.36 %, which may limit the application of the HMI system to samples with sufficiently high PNCs.

**Fig. 2:**
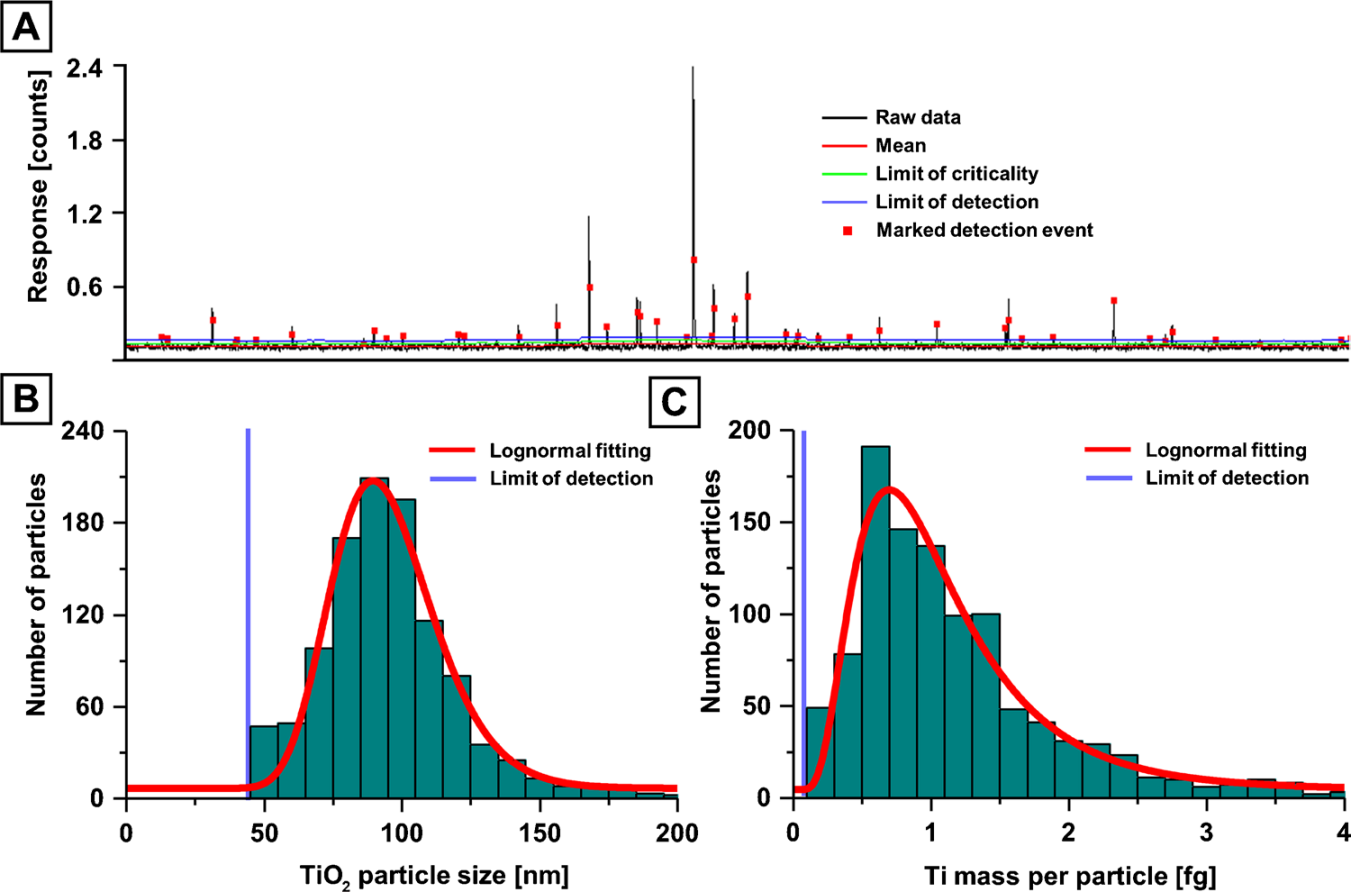
**A** SP ICP-MS/MS data monitoring ^48^Ti^16^O in a selected environmental sample. A Poisson filter was used to identify signals from NMs. **B** Detected signals were calibrated into TiO_2_ sizes and displayed in a histogram to visualise the size distribution. **C** Ti mass distribution across individual particles

**Fig. 3:**
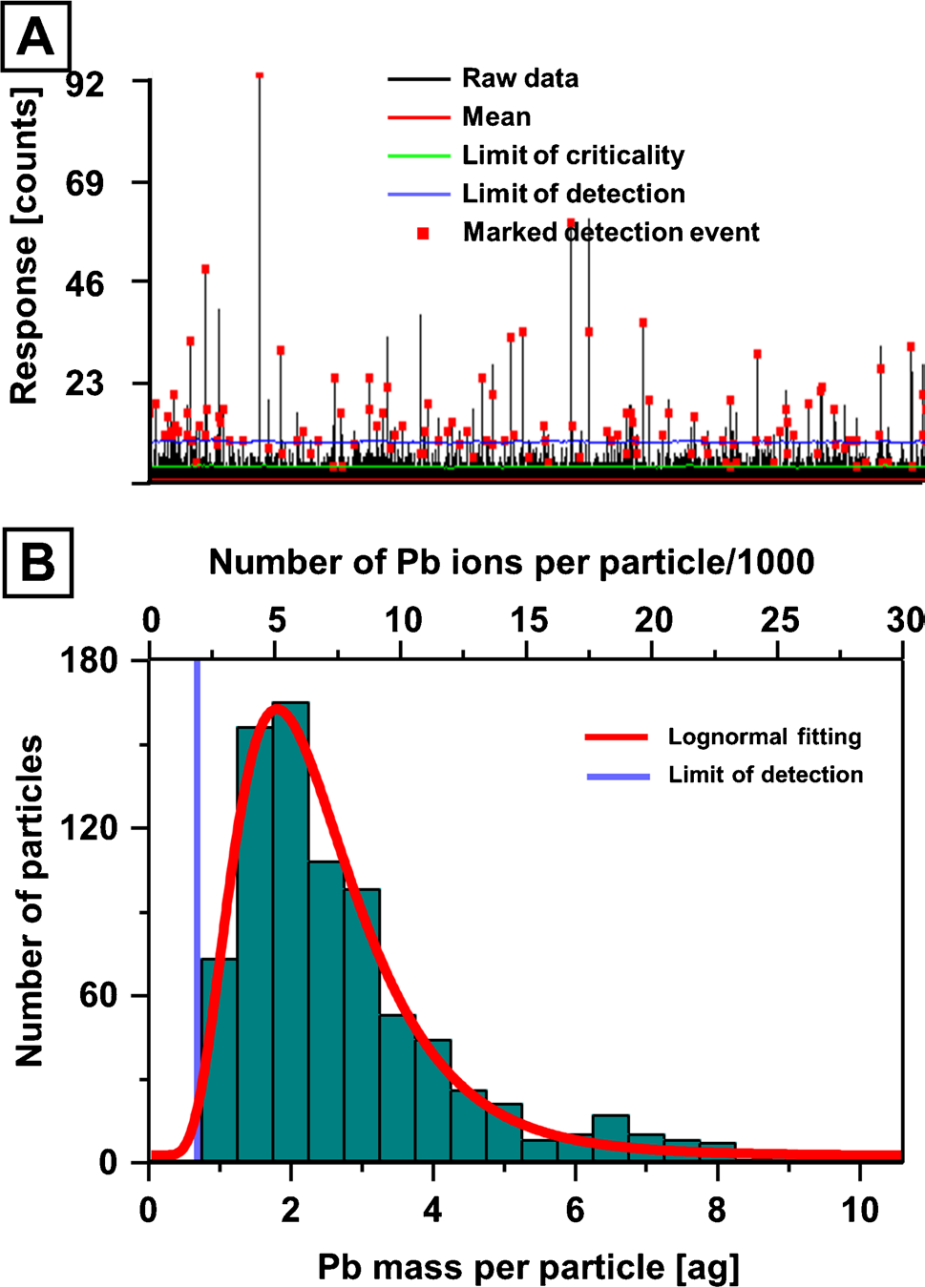
**A** Transient analysis of ^208^Pb. **B** Using the thresholding and calibration approach, signals were recognised and calibrated into a Pb mass distribution and the number of Pb ions per particle

Interferences for Ti isotopes were reduced by employing a mass shifting method and targeting ^48^Ti^16^O via SP ICP-MS/MS. Operating a mass analyser with short dwell times led to low background counting rates which could be modelled with a Poisson filter as previously discussed elsewhere [22, 26, 47]. Here, a dynamic Poisson filter was used to determine mean values across 1500 data points, from which limits of detection (blue line in Figure 2A) and criticality (green line in Figure 2A) were estimated based on Poisson statistics [46]. Signals exceeding the limits of detection (marked with red square) were summed for values above the limits of criticality and saved in a data array for size and mass calibration. In case of Ti, it was assumed that NMs were present as TiO_2_ to estimate the spherical diameter as shown in Figure 2B. Mass distributions may be calibrated as shown in Figure 2C. The screening method revealed a high abundance of Pb-containing NMs (Figure 3A). The properties of these NMs (e.g., density and Pb mass fraction) were unknown, and as such, mass calibration was preferred over size calibration. Two theories were considered for the occurrence of these NMs and are discussed in greater detail below; NMs may either consist of discrete insoluble Pb (e.g., PbO), which may originate from natural or incidental processes, or they may consist of ionic Pb accumulating on the surfaces of other nano-scaled particulate matter such as SiO_2_, TiO_2_, carbon or Al_2_O_3_. The latter theory was more plausible given that high levels of Pb were previously described in the investigated region and that ionic Pb tends to adsorb on sediments and larger particulate matter [48, 49]. The mass of Pb per particle can be calculated as shown in Figure 3B. The resulting distributions can further be translated into the number of Pb ions per NM as shown in the same figure but on the opposite *x*-axis. In this case, the PNC detection limit was assessed by analysing three blank solutions and applying the Poisson filter for signal recognition. The average background TiO_2_ and Pb particle count was 4.0 (± 2.9) and 5.3 (± 2.9) respectively, and only samples with significantly (3*σ*)  higher count rates were reported.

### Pb adsorption on nanomaterials

**Fig. 4:**
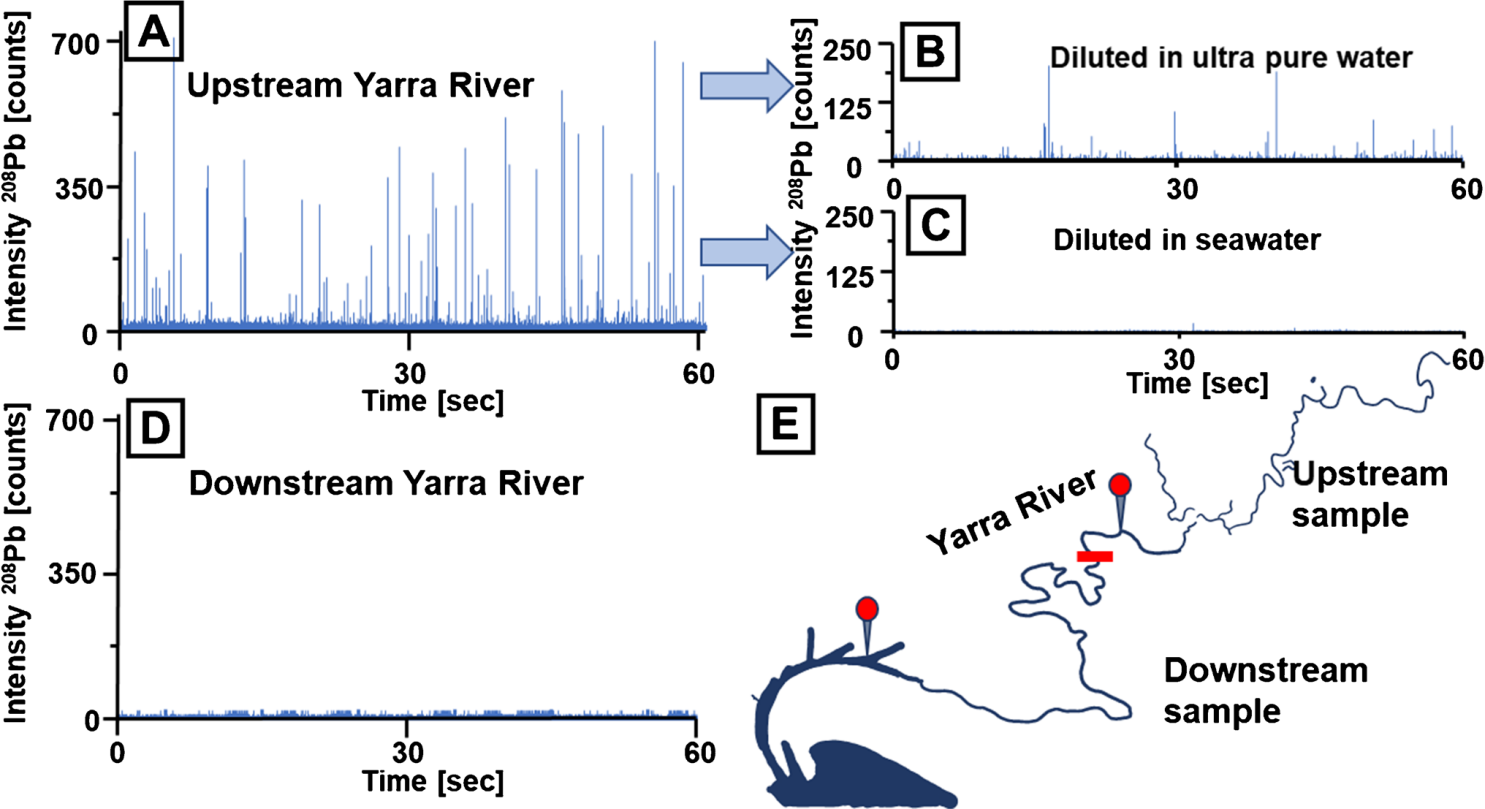
**A** SP ICP-MS raw data for the isotope ^208^Pb. Signal intensities decreased after dilution in **B** pure water and disappear in **C** seawater. **D** SP ICP-MS raw data for ^208^Pb in a sample containing brackish water. **E** Scheme of the Yarra River. Sampling locations and the weir are indicated

High levels of Pb have previously been described in soils and sediments in the metropolitan area of Melbourne [48, 50, 51] and are the legacy of urban development and industrial activities but may also be attributed to geogenic backgrounds. Previous studies have investigated the levels of Pb and its binding to particulate matter and demonstrated that a majority of Pb is bound to particles with sizes exceeding 400 nm [49, 52]. Nevertheless, little is known about the interaction of Pb with smaller nanoparticulate matter. One approach to investigate the adsorption of Pb to NMs is the application of flow-field flow fractionation coupled to ICP-MS as demonstrated by Loosli et al. [53]. However, if sufficient mass is accumulated, SP ICP-MS is applicable to study the adsorbed Pb across individual particles and to establish distribution models as demonstrated in this study. The adsorption of Pb on NMs was investigated after filtering samples with a 450 nm mesh to remove larger particles. To avoid the disruption of chemical equilibria, no further sample treatment was conducted and dilution was performed via the HMI system. This was crucial to control the introduction of environmental matrices into the plasma and to avoid signal drift. It was observed that Pb-containing NMs were most abundant in fresh water, with PNCs decreasing significantly as the freshwater mixed with salt water. Figure 4 shows the SP ICP-MS analysis of Pb-containing NMs in two representative samples obtained from the Yarra River system. Upstream, the Yarra River consists solely of freshwater but blends with seawater after flowing past a weir (Dights Falls, red bar in Figure 4E). Figure 4A and D compare the SP ICP-MS raw data for a selected pair of up- and downstream water samples (approximate location indicated in Figure 4E). While no discrete signals for Pb-containing NMs were detected in brackish water (downstream sample, Figure 4D), the freshwater sample (upstream sample, Figure 4A) contained several detectable signals corresponding to individual NMs. Sea- and brackish water have matrices with a high ion strength and may change the stability of dispersed NMs by altering their surface potentials and impacting the equilibrium between free and adsorbed ionic species. This may cause either the agglomeration of formerly stable NMs or the complete desorption of ionic Pb from the surface of NMs. To investigate the impact of seawater on the stability of Pb-containing NMs, the upstream sample was diluted in pure and seawater (1:2 v/v) as shown in Figure 4B and C, respectively. It was evident that the dilution in seawater resulted in the removal of Pb-containing NMs; however, they were still detectable after dilution in pure water. Interestingly, dilution in pure water did not only decrease the PNC, but also the signal intensity indicating that the dilution process caused a desorption of Pb from NMs. On the one hand, this supports the hypothesis that ionic Pb was adsorbed reversibly on nano-scaled particles, and on the other hand, it reinforces the requirement to apply aerosol dilution instead of matrix dilution.

The binding of Pb species to NMs is relevant for the mobility of Pb in the environment and may also have ecotoxicological implications. It has previously been shown that heavy metals may accumulate on vector NMs which then promote their bioavailability. For example, Wang et al. [54] demonstrated that the adsorption of Cd on TiO_2_ NMs has a transgenerational reproductive toxicity using *Caenorhabditis elegans* as model organism. Heavy metals may accumulate on the surface of NMs, desorb following ingestion by biota, bioaccumulate, and translocate within an organism. To investigate potential NM substrates, TEM/EDX was employed to investigate selected samples and identified TiO_2_ as well as SiO_2_-based particles as shown for two representative samples in Figures S3 and S4.

### Ti and Pb nanomaterials in the environment

The application of SP ICP-MS identified the presence of NMs in 78 % of the investigated samples, 76.5 % contained Ti-based NMs, and Pb-containing NMs were found in 53 % of the samples. As a general trend, it was observed that PNCs and mean Pb masses per particle decreased with increasing saltwater content. All calibrated PNCs, size, or mass distributions are listed with the corresponding ionic background concentration and the limits of detection in Table S2. One potential spectral interference that cannot be fully mitigated using an oxygen-based mass shifting methods was Ca (^48^Ca ➔ ^48^Ca^16^O) [55]. Consequently, the accuracy of determined ionic background may be impacted by high natural Ca levels. The mass detection limit for particulate Pb deviated from 0.8 to 5.7 ag and the size detection limit of TiO_2_ particles ranged from 24.4 to 72.5 nm depending on the ionic background levels. The highest PNCs detected were 395·10^6^ particles L^−1^ for Pb-containing NMs and 14,600·10^6^ particles L^−1^ for Ti-based NMs. The highest mean mass of particulate Pb was 117 ag and the largest detected mean size of TiO_2_ was 142 nm. The average size of TiO_2_ across all sampling locations was 79.8 nm, which was larger than sizes previously reported (typically between 40 and 50 nm) elsewhere [41, 53]. The TiO_2_ PNCs were generally within a range reported across various metropoles in a study by Azimzada et al. [34].

The emission of NMs from wastewater treatment plants (WWTPs) was investigated by sourcing water within the proximity of WWTPs’ effluents. In two cases, PNCs were compared up- and downstream of WWTP effluents. While in one case, the PNC decreased after the effluent, a significant increase was detected for the second effluent where Ti-based PNCs increased from no detected NMs to 3200 (± 116)·10^6^ particles L^−1^ and the Pb-containing PNCs increased from no detected NMs to 75.6 (± 6.1)·10^6^ particles L^−1^. Further samples were obtained within the proximity of effluents distributed across Melbourne, and the highest PNCs were calibrated to be 3440 (± 120)·10^6^ and 171 (± 9.1)·10^6^ particles L^−1^ for Ti- and Pb-containing NMs, respectively. Locations and further data are given in Table S2.

**Fig. 5:**
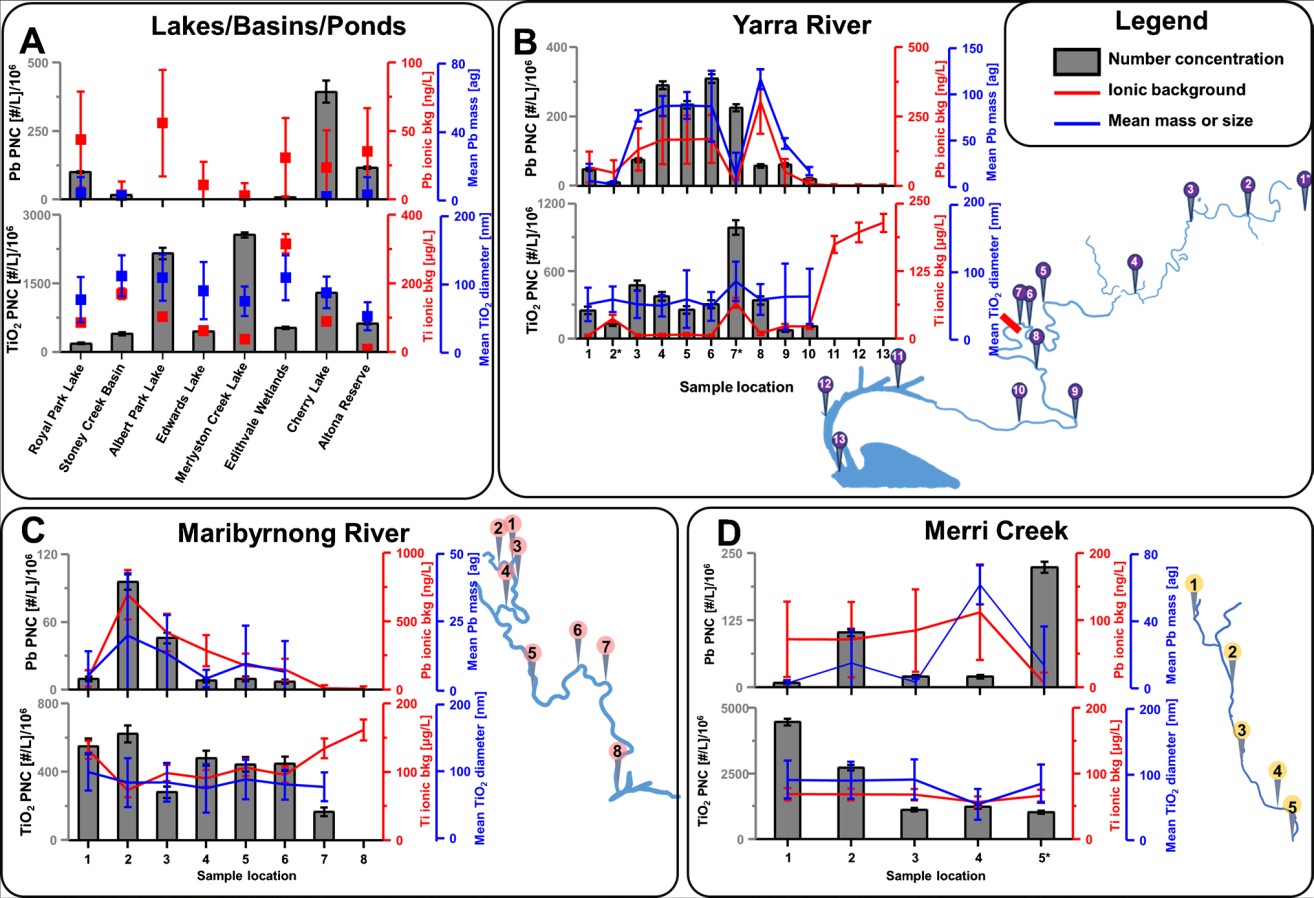
PNCs, mean sizes and masses, and the ionic background of TiO_2_ and Pb-containing NMs are shown across different sample locations of Melbourne. **A** Selected lakes and basins. **B** The Yarra River. Sample location 1 is not shown on the map and locations 2 and 7 were in the proximity of inflows of other rivers. **C** Maribyrnong River. **D** Merri Creek

There was no significant number of investigated NMs in samples obtained within the Port Phillip Bay however, in the Western Port, one sample returned the highest Ti PNC detected (14,600·10^6^ particles L^−1^) within this study. The origin of these NMs was not clear but potential sources may be a landfill and quarry within the proximity of the respective location. Waters from lakes, basins, and wetlands were analysed as shown in Figure 5A, and in all cases, Ti-based NMs were detected with PNCs ranging from 164 (± 26.3)·10^6^ to 2580 (± 104)·10^6^ particles L^−1^. Pb-containing NMs were found in three cases with PNCs of up to 395 (± 13.9)·10^6^ particles L^−1^. Figure 5B–D show the Ti (bottom) and Pb (top) PNCs, the mean masses/sizes, and the ionic background along the course of three streams in Melbourne. For comparisons, the geographic situation and the sampling locations are shown adjacently to each diagram. In case of the Yarra River (Figure 5B), samples were obtained at the margin of the metropolitan area (upstream) and downstream within the metropolitan area until the estuary located in the Port Phillip Bay (compare river scheme in Figure 5B). It was observed that over the course of the river, PNCs increased systematically within freshwaters. Outliers were caused by other rivers flowing into the Yarra River (locations 2 and 7 (marked with *)). The Yarra River is separated by a weir (Dight Falls, marked with red bar) into a freshwater section (upstream) and a section that is influenced by tidal seasons and consequently by saltwater (downstream). In the latter section, PNCs decreased substantially. A similar trend was observed for the Maribyrnong River (Figure 5C). For this river, PNCs and sizes of Ti-based NMs were relatively consistent upstream and decreased within the proximity of the Port Phillip Bay. Calibrated ionic Ti background levels were increased in seawater and may be influenced by high ionic Ca levels. Compared against river waters, Ca concentrations are significantly higher in seawater and potentially interfere with selective Ti analysis. Future studies may benefit from alternative mass shifting methods using more selective reaction gases to resolve ^48^Ca and ^48^Ti [56].

The highest Pb-containing PNCs were found upstream within the proximity of a major airport and decreased downstream towards Port Phillip Bay, where waters blended with seawater. Figure 5D shows the distribution of NMs across the course of Merri Creek, which is a smaller water stream that flows into the Yarra River shortly before the Dight Falls. Merri Creek transported the highest TiO_2_ PNC which was also recognisable at the Yarra River inflow (compare location 7, Figure 5B).

Urban environments are highly dynamic systems with a substantial anthropogenic pressure and further analysis is required in the future to predict the longitudinal and seasonal distribution, the size/mass of NMs as well as their impact on biota. Additionally, the application of SP ICP-ToF-MS may be advantageous to investigate the composition and origin of NMs. As such, the data reported here provides only a snapshot of PNCs, ionic background concentrations, and the masses/sizes of NMs. It is not clear whether the detected concentrations, masses, and sizes pose environmental hazards however, the adsorption of Pb on nanoparticulate matter may be relevant regarding its environmental mobility and bioavailability.

## Supplementary Information

Below is the link to the electronic supplementary material.

Supplementary file1 (PDF 357 kb)
