# Supplementary material for: Analysis of Ti- and Pb-based particles in the aqueous environment of Melbourne (Australia) via single particle ICP-MS
Source: Anal Bioanal Chem. 2022 Apr 28;414(18):5671–81. doi: 10.1007/s00216-022-04052-0 (PMC9242955; doi:10.1007/s00216-022-04052-0)
Supplement: Supplementary file 1 — Supplementary file1 (PDF 357 kb) [file 216_2022_4052_MOESM1_ESM.pdf]

Analysis of Ti and Pb-based Particles in the Aqueous Environment of Melbourne (Australia) via single particle ICP-MS

Raquel Gonzalez de Vega[1,2], Thomas E. Lockwood[2], Xiaoxue Xu[3], Claudia Gonzalez de Vega[2], Johannes Scholz[2,4], Maximilian Horstmann[2,4], Philip A. Doble[2], David Clases[1,2*]

[1] Institute for Chemistry, University of Graz, 8010 Graz, Austria

[2] The Atomic Medicine Initiative, University of Technology Sydney, 15 Broadway, Ultimo, NSW 2007, Australia

[3] School of Biomedical Engineering, University of Technology Sydney, 15 Broadway, Ultimo, NSW 2007, Australia

[4] University of Münster, Institute of Inorganic and Analytical Chemistry, Corrensstr. 480, 48149 Münster, Germany

***Corresponding author: email: David.Clases@uni-graz.at**

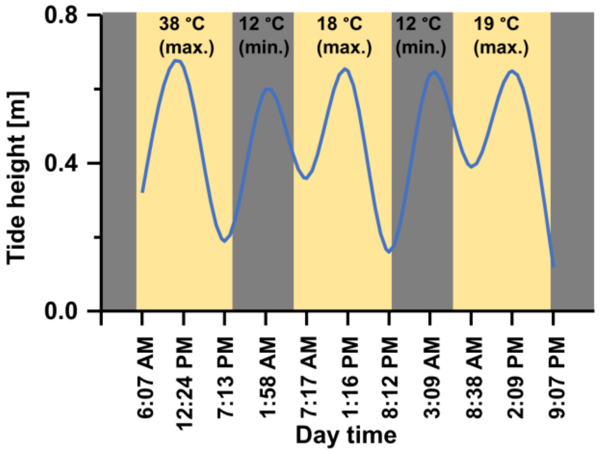

Figure S1. Temperature and tidal seasons during the sampling campaign.

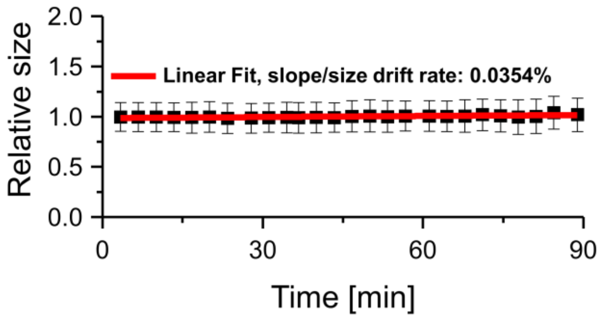

Figure S2. The robustness was investigated by repeatedly analysing and calibrating the size of Au NPs in seawater.

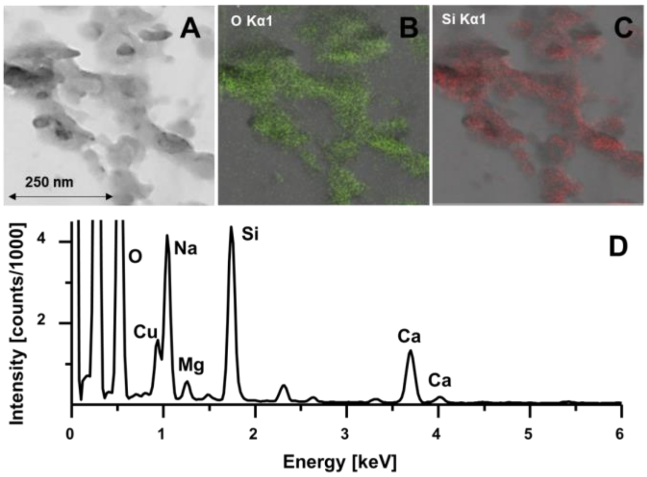

Figure S3. A: Representative nanostructures detected by TEM. **B** and **C** show the overlay of the TEM image with the O and Si distribution recorded via EDX (Kα1 emission), respectively. **D** shows the recorded energy dispersive spectrum (the Cu signal was caused by the sample support material).

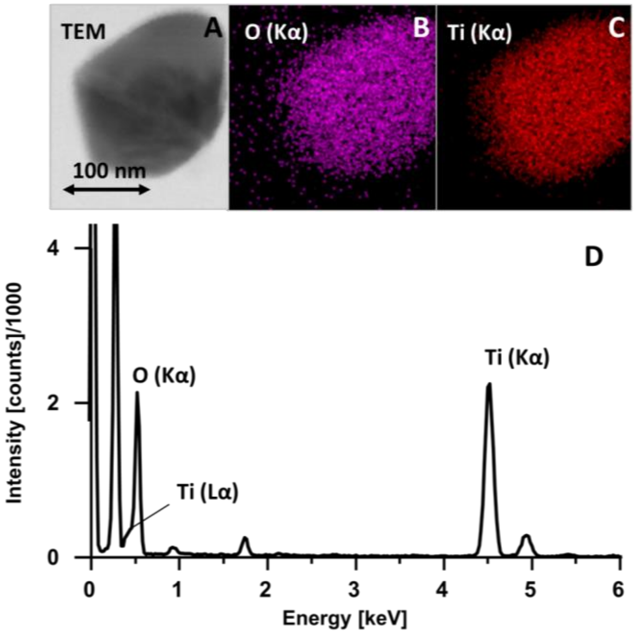

Figure S4. A: Representative nanostructure detected by TEM. **B** and **C** show the Kα1 emission lines of O and Ti recorded via EDX. **D** shows the recorded energy dispersive spectrum.

Table S1. Instrumental parameters

Parameter	Screening method	SP ICP-MS for Pb NMs	SP ICP-MS/MS for TiO_2
Mode	Single Quad	Single Quad	MS/MS
RF Power [W]	1600	1600	1600
Sampling Depth [mm]	4.0	4.0	4.0
Nebuliser flow [L/min]	0.70	0.70	0.70
Dilution Gas flow [L/min]	0.42	0.42	0.42
Extract 1 voltage [V]	-200.0	-200.0	-200.0
Extract 2 voltage [V]	-20.0	-10.0	-22.5
Omega Bias [V]	-200.0	-200.0	-170.0
Omega Lens [V]	17.0	13.0	11.0
Deflect [V]	20.0	20.0	10.0
SLG Factor	0.35	0.90	n/a
Q1 Mass Gain	3	127	127
OctP Bias [V]	-18.0	-30.0	-7.4
Axial Acceleration [V]	0.0	0.0	1.0
Energy Discrimin.a.tion [V]	1.5	0.0	-12.0
Oxygen cell gas flow [%]	0	0	20
Dwell time [ms]	0.1	0.1	0.1

Table S2. Sampling locations, descriptions, and calibrated data. When data is reported as not available (n.a.) for some samples, initial screening did not find any particles and therefore, samples were not further analysed or calibrated.

Sample group/ID/analyte	PNC [#/L] $\cdot 10^6$	Pb mean mass [ag]<br>Ti mean size [nm]	Ionic bkg [ng Pb/L]<br>[µg Ti/L]	Pb mass LOD [ag]<br>Ti size LOD [nm]	Approx. sample location, date and time	Sample description
**Yarra River**						
Y1, Pb<br>Y1, TiO_2	43.6±4.6<br>240±31.6	6.9±18.0<br>52.3±25	67.2±55.1<br>4.8±2.0	1.5<br>25.0	-37.735899, 145.221362<br>11.12.2019, 10:50	Warrandyte Federation Playspace
Y2, Pb<br>Y2, TiO_2	5.3±1.6<br>122±22.6	3.1±3.1<br>59.6±18.9	46.2±45.2<br>36.5±6.9	1.2<br>40.5	-37.753549, 145.118251<br>10.12.2019, 10:10	Ruffey Creek inflow
Y3, Pb<br>Y3, TiO_2	70.7±5.9<br>462±44.1	76.7±6.2<br>52.3±20.1	131±76.7<br>5.3±2.2	3.3<br>25.8	-37.757005, 145.074195<br>10.12.2019, 09:07	Possum Hollow Playground
Y4, Pb<br>Y4, TiO_2	290±11.9<br>366±39.2	87.5±10.9<br>50.3±15.8	166±87.5<br>6.3±2.4	5.2<br>26.4	-37.780986, 145.062152<br>10.12.2019, 08:47	Main Yarra Trail Bridge
Y5, Pb<br>Y5, TiO_2	233±10.6<br>244±32.0	88.0±14.5<br>60±42.4	167±88.0<br>7.4±2.6	5.5<br>28.5	-37.785371, 145.013241<br>10.12.2019, 08:16	Fairfield Pipe Bridge
Y6, Pb<br>Y6, TiO_2	310±12.3<br>294±35.2	87.3±38.3<br>49.2±21	169±87.3<br>4.8±2.1	3.4<br>25.2	-37.795856, 145.002345<br>09.12.2019, 18:15	Before Merri Creek inflow
Y7, Pb<br>Y7, TiO_2	224±10.4<br>980±64.2	13.6±23.4<br>86.4±29.1	5.6±16.2<br>66±8.9	0.9<br>46.3	-37.795626, 145.001637<br>09.12.2019, 18:20	Merri Creek inflow
Y8, Pb<br>Y8, TiO_2	53.8±5.1<br>328±37.1	117±10.8<br>59.5±22.7	303±117<br>9.3±3.0	5.7<br>28.7	-37.809705, 145.009435<br>09.12.2019, 17:52	Dickinsons Reserve
Y9, Pb<br>Y9, TiO_2	57.2±5.3<br>63.1±16.3	47.1±5.7<br>63.5±49.2	49.7±47.1<br>22.5±4.7	2.6<br>35.6	-37.826824, 145.022832<br>09.12.2019, 17:33	Wurundjeri Gardens
Y10, Pb<br>Y10, TiO_2	15.5±2.7<br>96.7±20.2	17.1±4.6<br>64.0±41.0	6.7±17.1<br>22.2±4.7	2.7<br>35.5	-37.832121, 145.003971<br>09.12.2019 17:02	Herring Island

Y11, Pb<br>Y11, TiO_2	n.a.<br>n.a.	n.a.<br>n.a.	0.56±4.9<br>174±15.7	n.a.<br>60.8	-37.818017, 144.932560<br>09.12.2019, 14:05	Bolte Bridge
Y12, Pb<br>Y12, TiO_2	n.a.<br>n.a.	n.a.<br>n.a.	0.74±5.2<br>197±18.3	n.a.<br>63.6	-37.828306, 144.897870<br>09.12.2019, 15:00	Fishermans Bend
Y13, Pb<br>Y13, TiO_2	n.a.<br>n.a.	n.a.<br>n.a.	0.73±5.6<br>214±16.7	n.a.<br>65.2	-37.847137, 144.898492<br>11.12.2019, 07:40	Port Philip Estu-<br>ary
**Maribyrnong River**						
MA1, Pb<br>MA1, TiO_2	9.2±2.1<br>559±48.5	5.6±8.8<br>99.2±27.9	82.4±60.4<br>132±13.4	1.5<br>56.7	-37.701234, 144.838915<br>09.12.2019, 09:09	Arundel Creek
MA2, Pb<br>MA2, TiO_2	95.0±6.8<br>635±51.7	20.2±22.8<br>83.1±36.8	695±180<br>73.5±10.6	5.3<br>48.2	-37.704458, 144.832450<br>09.12.2019, 09:17	Arundel Rd<br>Bridge
MA3, Pb<br>MA3, TiO_2	45.5±4.7<br>282±34.4	13.6±14.2<br>83.6±29.0	418±137<br>98.4±11.8	3.9<br>52.0	-37.717806, 144.842246<br>09.12.2019, 09:35	Caroline<br>Chisholm Park
MA4, Pb<br>MA4, TiO_2	7.8±1.9<br>488±45.3	4.4±3.2<br>74.4±36.4	285±112<br>90.6±12.2	3.0<br>51.1	-37.727058, 144.836210<br>09.12.2019, 10:05	Garden Ave
MA5, Pb<br>MA5, TiO_2	9.2±2.1<br>450±43.5	9.9±14.0<br>87.8±29.8	176±87.9<br>106±12.2	2.4<br>53.2	-37.770223, 144.851616<br>09.12.2019, 11:15	Solomon's Ford
MA6, Pb<br>MA6, TiO_2	6.8±1.8<br>454±43.7	7.0±11.2<br>80.2±22.3	147±80.6<br>95.9±11.6	2.2<br>51.5	-37.751484, 144.879715<br>09.12.2019, 11:40	Steele Creek in-<br>flow
MA7, Pb<br>MA7, TiO_2	n.a.<br>164±26.3	n.a.<br>76.6±21.6	10.4±21.2<br>134±14.2	1.0<br>55.5	-37.764425, 144.898559<br>09.12.2019, 12:00	Ladies Cricket<br>Club
MA8, Pb<br>MA8, TiO_2	n.a.<br>n.a.	n.a.<br>n.a.	6.8±16.6<br>161±15.3	0.9.<br>58.9.	-37.812114, 144.906321<br>09.12.2019, 14:42	Yarra River inflow
**Merri Creek**						
ME1, Pb<br>ME1, TiO_2	7.3±1.9<br>980±64.2	3.0±1.9<br>86.4±29.1	71.5±56.1<br>66.0±8.9	1.5<br>46.3	-37.728780, 144.965997<br>09.12.2019, 19:30	Before Sanne's<br>Bend
ME2, Pb<br>ME2, TiO_2	102±7.0<br>1180±70.5	15.1±19.4<br>54.7±23.6	71.1±56.3<br>56.6±7.9	1.5<br>45.0	-37.742437, 144.979038<br>09.12.2019, 19:15	Highway 40<br>Bridge
ME3, Pb<br>ME3, TiO_2	18.4±3.0<br>1080±67.3	3.6±3.0<br>92.6±30.5	84.6±61.6<br>67.7±8.9	1.6<br>47.2	-37.772573, 144.985658<br>09.12.2019, 18:47	Merri Park
ME4, Pb<br>ME4, TiO_2	18.4±3.0<br>2680±106	61.8±11.5<br>91.1±28.3	112±71.0<br>68.0±9.1	1.9<br>47.5	-37.783680, 145.001010<br>09.12.2019, 18:35	George Knott Re-<br>serve
ME5, Pb<br>ME5, TiO_2	224±10.4<br>4430±248	13.6±23.4<br>92.2±28.8	5.6±16.2<br>68.3±9.5	0.9<br>48.3	-37.795626, 145.001637<br>09.12.2019, 18:20	Yarra River inflow<br>(same as Y7)
**Mooney Ponds Creek**						
MO1, Pb<br>MO1, TiO_2	8.2±2.0<br>479±44.9	7.7±8.8<br>95.2±47.7	91.5±64.0<br>81.5±10.5	1.7<br>48.5	-37.742351, 144.932058<br>09.12.2019, 12:30	Before M2 bridge
MO2, Pb<br>MO2, TiO_2	7.5±1.9<br>526±47.0	7.3±8.1<br>82.1±26.1	76.7±58.3<br>65.0±9.0	1.5<br>46.6	-37.784819, 144.939538<br>09.12.2019, 13:00	Under M2 bridge
MO3, Pb<br>MO3, TiO_2	n.a.<br>164±26.3	n.a.<br>89.4±24.3	5.7±16.2<br>160±19.9	n.a.<br>59.9	-37.817250, 144.932232<br>09.12.2019, 14:00	Yarra River inflow

**Port Philip Bay**						
PP1, Pb<br>PP1, TiO_2	n.a.<br>n.a.	n.a.<br>n.a.	n.a.<br>188±73.5	n.a.<br>62.2	-37.850465, 144.950317<br>09.12.2019, 16:15	Kerferd Road Pier
PP2, Pb<br>PP2, TiO_2	n.a.<br>n.a.	n.a.<br>n.a.	n.a.<br>142±13.2	n.a.<br>56.9	-38.011487, 145.086592<br>10.12.2019, 13:30	Mordialloc Fore-shore Reserve
PP3, Pb<br>PP3, TiO_2	n.a.<br>n.a.	n.a.<br>n.a.	n.a.<br>150±15.3	n.a.<br>57.7.	-38.305517, 144.680971<br>10.12.2019, 18:00	Point Nepean Cemetery
PP4, Pb<br>PP4, TiO_2	n.a.<br>n.a.	n.a.<br>n.a.	n.a.<br>200±18.0	n.a.<br>62.6	-38.308341, 145.198454<br>10.12.2019, 19:25	Fred Smith Re-serve
PP5, Pb<br>PP5, TiO_2	n.a.<br>n.a.	n.a.<br>n.a.	n.a.<br>200±20.0	n.a.<br>62.9	-37.875438, 144.813913<br>11.12.2019, 09:30	Apex Park
PP6, Pb<br>PP6, TiO_2	n.a.<br>n.a.	n.a.<br>n.a.	n.a.<br>189±20.0	n.a.<br>60.8	-38.005333, 144.590688<br>12.12.2019, 11:45	Little River estu-ary
PP7, Pb<br>PP7, TiO_2	n.a.<br>n.a.	n.a.<br>n.a.	n.a.<br>193±23.0	n.a.<br>61.8	-38.027819, 144.565216<br>11.12.2019, 13:10	Point Wilsons Boat Ramp
PP8, Pb<br>PP8, TiO_2	9.7±2.2<br>14600±248	3.3±2.8<br>142±44.4	1.0±6.8<br>227±46.4	0.8<br>70.4	-38.406324, 145.527160<br>12.12.2019, 14:00	Granville Fore-shore Reserve
**Western streams**						
WC1, Pb<br>WC1, TiO_2	n.a.<br>261±33.1	n.a.<br>83.9±39.7	30.8±37.1<br>78.5±9.7	1.2<br>49.2	-37.989232, 145.106063<br>10.12.2019, 12:50	Canal next to river
WC2, Pb<br>WC2, TiO_2	n.a.<br>n.a.	n.a.<br>n.a.	1.3±8.0<br>185±19.1.	0.8<br>58.6	-38.010289, 145.087053<br>10.12.2019, 13:20	Estuary
WC3, Pb<br>WC3, TiO_2	n.a.<br>198±28.8	n.a.<br>90.9±46.6	39.1±41.4<br>76.8±9.6	1.3<br>48.7	-38.030075, 145.182539<br>10.12.2019, 14:00	Gaelic Park
WC4, Pb<br>WC4, TiO_2	19.9±3.1<br>572±49.0	3.9±6.9<br>93.2±24.0	42.2±43.1<br>72.9±9.3.	1.2<br>47.5	-38.031503, 145.185968<br>10.12.2019, 14:10	Dandenong Creek
WC5, Pb<br>WC5, TiO_2	n.a.<br>1410±76.9	n.a.<br>76.5±20.8	4.5±14.0<br>43.5±7.1	0.8<br>41.7	-38.031429, 145.184453<br>10.12.2019, 14:20	Mordialloc/Dan-denong Cr. fork
WC6, Pb<br>WC6, TiO_2	n.a.<br>244±32.0	n.a.<br>78.4±26.4	8.2±18.7<br>49.9±7.3	0.9<br>42.3	-38.031139, 145.184111<br>10.12.2019, 14:33	Mordialloc/Dan-denong Cr. fork
WC7, Pb<br>WC7, TiO_2	55.2±5.2<br>n.a.	4.7±5.3<br>n.a.	71.2±57.1<br>178±20	1.5<br>61	-38.081744, 145.127321<br>10.12.2019, 14:55	Kananook Creek
**Lakes/Ponds/ Basins**						
L1, Pb<br>L1, TiO_2	96.4±6.8<br>164±26.3	7.0±14.7<br>77.6±32.9	67.7±55.1<br>85.6±10.1	1.5<br>48.1	-37.782784, 144.940261<br>09.12.2019, 13:10	Royal Park Lake
L2, Pb<br>L2, TiO_2	10.7±2.3<br>378±39.8	4.5±4.0<br>112.5±29.9	5.3±15.1<br>169±15.5	0.9<br>60	-37.828084, 144.895817<br>09.12.2019, 15:00	Stoney Creek Ba-sin
L3, Pb<br>L3, TiO_2	n.a.<br>2170±95.5	n.a.<br>110±33.5	86.2±61.5<br>102±11.5	1.7<br>51.5	-37.841931, 144.964705<br>09.12.2019, 16:35	Albert Park Lake
L4, Pb<br>L4, TiO_2	n.a.<br>428±42.5	n.a.<br>90.8±41.7	16.3±26.5<br>61.6±8.1	1.0<br>46.7	-37.713653, 144.990825<br>09.12.2019, 19:55	Edwards Lake

L5, Pb	n.a.	n.a.	4.6±14.0	0.8	-37.691027, 144.936998	Merrylston Creek
L5, TiO_2	2580±104	75.4±21.5	36.5±6.4	40.3	09.12.2019, 20:10	Lake
L6, Pb	n.a.	n.a.	47.4±45.5	1.3	-38.033532, 145.122820	Edithvale Wet-
L6, TiO_2	513±46.4	110±32.8	316±28.0	72.5	10.12.2019, 13:44	lands
L7, Pb	395±13.9	4.1±3.1	36.0±43.1	1.2	-37.861660, 144.833663	Cherry Lake
L7, TiO_2	1290±73.8	88.3±23.1	88.6±10.4	50.8	11.12.2019, 08:20	
**Wastewater Treatment Plants**						
WW1, Pb	n.a.	n.a.	n.a.	n.a.	-38.004969, 144.589780	Lake Borrie
WW1, TiO_2	84.1±18.8	65.5±21.6	71.2±11.1	46.0	11.12.2019, 12:10	
WW2, Pb	n.a.	n.a.	n.a.	n.a.	-38.004620, 144.591669	Little River estu-
WW2, TiO_2	n.a.	n.a.	122±16.7	54.7	11.12.2019, 12:20	ary
WW3, Pb	n.a.	n.a.	35.5±39.6	1.2	-38.010704, 144.581074	Effluent
WW3, TiO_2	130±23.4	85.7±43.9	81.6±9.9	48.2	11.12.2019, 12:40	
WW4, Pb	25.2±3.5	39.6±5.2	4.2±4.0	1.3	-38.015670, 144.576954	Effluent
WW4, TiO_2	3440±120	88.5±17.6	88.8±11.1	50.2	11.12.2019, 12:55	
WW5, Pb	n.a.	n.a.	n.a.	n.a.	-37.873638, 144.805858	Pond close to
WW5, TiO_2	75.7±17.8	123±53.0	86.4±17.2	50.1	11.12.2019, 09:00	WWTP
WW6, Pb	n.a.	n.a.	n.a.	n.a.	-37.873965, 144.804913	Pond close to
WW6, TiO_2	88.3±19.3	57.7±21.7	25.7±5	36.2	11.12.2019, 09:05	WWTP
WW7, Pb	171±9.1	3.9±2.4	20.6±30.8	1.0	-37.874312,144.802843	Effluent
WW7, TiO_2	303±35.7	52.0±24.2	4.1±2	24.4	11.12.2019, 09:11	
WW8, Pb	113±7.4	5.45±16.1	54.1±50.1	1.4	-37.874626, 144.807724	Altona Reserve
WW8, TiO_2	605±50.4	53.6±20.6	7.6±2.6	27.7	11.12.2019, 09:17	
WW9, Pb	n.a.	n.a.	24.1±32.3	1.0	-38.087554, 145.505528	Creek before
WW9, TiO_2	n.a.	n.a.	n.a.	n.a.	12.12.2019, 12:38	WWTP
WW10, Pb	75.6±6.1	3.5±4.7	21.5±31.2	1.0	-38.124115, 145.463903	Creek after
WW10, TiO_2	3200±116	90.4±35.6	86.1±13.2	51.2	12.12.2019, 12:15	WWTP
WW11, Pb	n.a.	n.a.	27.7±34.8	1.0	-37.755050, 145.291948	Brushy Creek, be-
WW11, TiO_2	1710±84.7	87.9±39.4	122±16.9	60.7	10.12.2019, 11:40	fore WWTP
WW12, Pb	n.a.	n.a.	46.2±44.7	1.2	-37.770822, 145.298570	Brushy Creek, af-
WW12, TiO_2	1320±74.5	81.4±25.2	113±15.8	54.0	10.12.2019, 11:25	ter WWTP
WW13, Pb	n.a.	n.a.	n.a.	n.a.	-38.440328, 144.847840	Boags Rocks, ef-
WW13, TiO_2	n.a.	n.a.	n.a.	n.a.	10.12.2019, 16:34	fluent
